# Gynecological Clinique

**Published:** 1871-09

**Authors:** De Laskie Miller


ARTICLE V.—*Gynecological Clinique. North Chicago Charity Dispensary. Rush Medical College.* Every Thursday. Prof. DE LASKIE MILLER. Reported by C. T. FENN, M.D., Chicago, Ill.

Two flights of stairs to the right of the north entrance to the College lead to the patients' waiting room. Ventilated, light and warm, provided with settees, a sink and a water closet. Adjoining the waiting room are two apartments: one, a small room for the examination of children and such of the women as do not require special treatment; the other, a commodious and beautiful room, furnished with everything essential in the conduct of the Clinique. A low operating table, mattress and pillow occupy the centre, fronted by two large windows, whose light is reflected from all sides by white walls and an oblique ceiling. A sofa, some chairs, a table, a sink, a closet with drawers, and a screen before the door, complete the furniture. Another entrance opposite that from the waiting room opens from the College library room.

Delicacy and kindness are always necessary in the treatment of the indigent; but, the sensibility being often inversely as the refinement, it requires the highest degree of tact to induce all who apply to this department to subserve the interests of medical instruction. While they are received and treated with the deference due to womanhood, they are required to obey the rules. Convenience and efficiency are promoted by pretty strict discipline on the part of medical attendants, patients and visitors.

Every patient who comes is registered by name, age, nativity, residence, occupation, condition as to marriage, number of children, miscarriages, and date of confinements; previous and present complaints; supposed causes; menstruation, regularity, amount, suffering; leucorrhœa, regularity, amount, constancy; pain, regularity, constancy; locomotion; other symptoms, digestive, and physical signs. Examinations of the uterine organs are made by touch, speculum and probe, whenever necessary. The diagnosis and the treatment are recorded at each visit. The other entries are made but once. No routine is followed in the treatment. Anæmic, tuberculous or syphilitic conditions are met by appropriate remedies as often as they are present. Each patient receives a card containing a name and corresponding number.

The management has proved both easy and satisfactory. It is an invariable custom to protect the person. Whenever a patient reclines in the course of an examination the portions of her body liable to exposure are covered with a spread. A thick veil is provided also, which she may draw over her face. With an assurance of strict obedience to this precaution, patients are rarely much affected by the presence of others.

During the hour appointed, students, in classes of ten, are admitted by an assistant in charge of the library door. They are permitted to witness the modes of diagnosis and of treatment, are carefully instructed in what pertains to the case, and then are ushered out the way they entered. An assistant waits on the patients, having them in readiness. A third assistant keeps the books and prepares prescriptions. Another well trained assistant waits on the gynecological attendant.

It is proposed hereafter to report a few cases taken among the patients as they came, without regard to special interest. They will present a fair average of their class. The present effort is to point out the advantages of the Dispensary, and to indicate some features of its management.

The following table shows the number and character of cases admitted from February 24, to July 28, 1870, twenty-three weeks. They have been followed by many more, the average attendance being now over twenty each week.

| Number. | Nativity. | Age. | Occupation. | Married or Single. | No. of Children. | No. of Abortions. | Time since last Confinement. | Duration of Sickness. | Date of Admission. | DIAGNOSIS. |
|---|---|---|---|---|---|---|---|---|---|---|
| 1 | Ger'y | 22 | Domestic. | S. | 1 | | 18 m | | Feb. 24 | Vaginitis. |
| 2 | Ill. | 17 | Seamstress. | S. | | | | 3 y | " " | Dysmenorrhœa and constipation. |
| 3 | Irel'd | 29 | Housewife. | M. | 2 | | 2 y | | " " | Vaginitis and metritis. |
| 4 | " | 36 | " | M. | 6 | | 10 m | 6 m | " " | Metritis and constipation. |
| 5 | | 18 | | S. | | | | | " " | Amenorrhœa and constipation. |
| 6 | N. Y. | 38 | Seamstress. | S. | | | | | " " | Chr. metritis and displacement. |
| 7 | Irel'd | 33 | Housewife. | M. | None | | | 8 y | " " | Chr. metritis and retroversion. |
| 8 | Eng. | 39 | " | M. | 6 | 2 | 12 y | 4 y | " " | Cervical endometritis. |
| 9 | Irel'd | 31 | " | M. | None | 5 | 5 y | | " " | Vaginitis and metritis. |
| 10 | " | 38 | Nurse. | M. | 6 | 2 | 5 y | 2 y | " " | Menorrhagia. |
| 11 | " | 28 | Domestic. | S. | | | | 6 m | Mar. 3 | Dysmenorrhœa. |
| 12 | " | 27 | Housewife. | M. | None | | | 9 y | " " | Chr. metritis. |
| 13 | Eng. | 44 | " | M. | 9 | | | 3 y | " " | Ovarian tumor. |
| 14 | | 13 | School. | | | | | 1 m | " " | Anæmia. |
| 15 | | 45 | Housewife. | M. | 4 | 1 | 4 y | | " " | Vaginitis. |
| 16 | Irel'd | 25 | " | M. | 3 | | 19 m | 1 y | " 10 | Ovarian tumor cyst. |
| 17 | City. | 3 | | | | | | 3 y | " " | Chronic meningitis. |
| 18 | Penn. | 36 | Housewife. | M. | 2 | | 4 y | 6 m | " 17 | Endometritis and ulcer. |
| 19 | Mass. | 23 | " | M. | None | | | 2 m | " 24 | Engorgement uteri. |
| 20 | N. Y. | 38 | " | M. | | 2 | 2 m | 2 m | " " | Delayed involution. |
| 21 | Scot. | 42 | " | M. | 9 | | 5 y | 6 m | " " | Dysmenorrhœa. |
| 22 | City. | 3 m | | | | | | 3 w | Apr. 7 | Ton contrac. lig. nuchæ. |
| 23 | Irel'd | 33 | Housewife. | M. | None | | | 6 y | " " | Chr. metritis. |
| 24 | " | 25 | " | M. | None | | | 18 m | " " | Chr. metritis. |
| 25 | N. Y. | 11 | School. | | | | | 2 y | " " | Hemorrhage of bowels. |
| 26 | Irel'd | 35 | Housewife. | M. | 1 | | 12 y | 12 y | " " | Metritis and displacement. |
| 27 | Ger'y | 59 | | M. | | | 20 y | | " " | Menorrhagia. |
| 28 | Irel'd | 26 | Domestic. | M. | | 1 | 4 y | 1 y | " " | Metritis and displacement. |
| 29 | " | 17 | " | S. | | | | 4 y | " " | Dysmenorrhœa. |
| 31 | " | 28 | Housewife. | M. | 5 | | 2 m | 2 m | " " | Defective involution. |
| 32 | " | 40 | Domestic. | S. | | | | 4 m | " " | Amenorrhœa. |
| 33 | " | 21 | | M. | | | | | " " | Amenorrhœa. |
| 34 | " | 26 | Housewife. | M. | 5 | | 7 m | 3 y | " " | Prolapsus of bladder. |
| 35 | " | 18 | Domestic. | S. | | | | 3 m | " 14 | Anæmia. |
| 36 | Swed. | 54 | Housewife. | M. | | | | 1 y | " " | Vaginitis. |
| 37 | Irel'd | 38 | " | M. | 3 | | | 2 m | " " | Vaginitis. |
| 38 | N. Y. | 30 | " | M. | None | | | 2 y | " " | Endometritis and retroversion. |
| 39 | Irel'd | 28 | " | M. | 4 | | 4 m | 4 m | " " | Chr. metritis. |
| 40 | Eng. | 39 | " | M. | 8 | 4 | 10 w | 2 y | " " | Menorrhagia. |
| 41 | Ger'y | 42 | " | M. | 3 | | 14 y | 14 y | " 21 | Chr. metritis. |
| 30 | Irel'd | 45 | " | M. | 1 | 2 | 6 y | 8 y | " " | Amenorrhœa. |
| 31 | " | 40 | " | M. | 6 | | 4 y | 4 y | " 28 | Engorgement and prolapsus. |
| 32 | " | 28 | " | M. | 2 | | 21 m | 3 m | " " | Endometritis. |
| 33 | " | 28 | " | M. | 2 | | 2 y | 6 m | May 5 | Endometritis. |
| 34 | City. | 2 | | | | | | 3 w | " " | Inflammatory diarrhœa. |
| 35 | Eng. | 14 | School. | | | | | | " " | Dysmenorrhœa. |
| 36 | N. Y. | 24 | Housewife. | M. | 1 | 6 | 10 m | 4 m | " 19 | Pregnancy. |
| 37 | | | " | M. | 6 | 2 | 8 m | | " " | Chr. metritis. |
| 38 | Irel'd | 19 | Dressmaker | S. | | | | 5 m | " " | Amenorrhœa. |
| 39 | " | 37 | Housewife. | M. | 7 | 2 | 19 m | 7 y | " " | Engorgement & excoriated cervix. |
| 40 | | 4 | | | | | | | " " | |
| 41 | S. C. | 12 | School. | | | | | 11 y | " " | Ichthyosis. |
| 42 | Irel'd | 44 | | M. | 6 | | 3 y | 3 y | " " | Chr. engorgement. |
| 43 | " | 40 | | M. | 3 | | 12 y | 9 y | " " | Chr. metritis and displacement. |
| 44 | | 8 | School. | | | | | | " 26 | Pleuritis circumscribed. |
| 45 | Irel'd | 44 | Housewife. | M. | 4 | 2 | 6 y | | " " | Chr. metritis. |
| 46 | " | 25 | | M. | | | | | " " | Delayed menstruation. |
| 47 | " | 22 | Domestic. | S. | | | | 18 m | " " | Dysmenorrhœa. |
| 48 | " | 50 | Housewife. | | | | | | " " | Chr. metritis. |
| 49 | Ca. | 28 | | M. | None | | | 4 y | June 2 | Endocervicitis. |
| 50 | N. Y. | 23 | Housewife. | M. | 1 | | 4 m | 4 m | " " | Defective involution. |
| 51 | Irel'd | 40 | " | M. | 7 | 2 | 16 m | 5 y | " " | Endocervicitis. |
| 52 | " | 38 | " | M. | 3 | 3 | | | " 9 | Chr. metritis. |
| 53 | " | 45 | " | M. | 2 | 7 | 10 d | 12 y | " " | Complete prolapsus. |

| Number. | Nativity. | Age. | Occupation. | Married or Single. | No. of Children. | No. of Abortions. | Time since last Confinement. | Duration of sickness. | Date of Admission. | DIAGNOSIS. |
|---|---|---|---|---|---|---|---|---|---|---|
| 54 | Irel'd | 45 | Housewife. | M. | 9 | | | 3 y | June 9 | Ulceration of cervix. |
| 55 | Ger'y | | | | 3 | | 3 y | 3 y | " 16 | Chr. metritis. |
| 56 | Irel'd | 40 | Housewife. | M. | 5 | 3 | 2 m | | " " | Cervicitis. |
| 57 | | | | S. | | | | | " " | Anæmia. |
| 58 | Irel'd | 37 | | M. | None | | | 4 y | " 23 | Endocervicitis. |
| 59 | " | 46 | Housewife. | M. | 8 | | 3 y | 6 y | " 30 | |
| 60 | " | 28 | | M. | 5 | 1 | 3 y | 1 y | " " | |
| 61 | Ger'y | 29 | | M. | 3 | | 8 y | | " 23 | |
| 62 | Eng. | 15 | | S. | | | | 3 y | " 30 | Delayed menstruation. |
| 63 | | 28 | | M. | 1 | | 10 m | | " " | Endocervicitis and retroversion. |
| 64 | City. | 5 m | | | | | | | July 7 | Psoriasis capitis. |
| 65 | Irel'd | 40 | | M. | 3 | | 8 y | | " 14 | Amenorrhœa. |
| 66 | | 40 | | M. | 5 | | 2 y | | " " | |
| 67 | | | | | | | | | " " | Debility. |
| 68 | N. Y. | 2 | | | | | | | " " | Worms. |
| 69 | Irel'd | 35 | Housewife. | M. | 2 | | 6 w | | " " | Debility. |
| 70 | " | 30 | | M. | 2 | | 6 y | 3 y | " " | Chr. metritis. |
| 71 | | 32 | Housewife. | M. | 6 | | 3 y | 6 y | " " | Enlargement of womb and ulcer. |
| 72 | | 16 m | | | | | | | " 21 | Chr. diarrhœa. |
| 73 | Eng. | 30 | Housewife. | M. | 2 | | 3 m | 16 m | " " | Prolaps. & ulcer uterus & vagina. |
| 74 | | 3 | | | | | | | " " | Dysentery. |
| 75 | Asia. | 26 | Housewife | M | 1 | 1 | 4 m | | " " | Endocervicitis and ulcer. |
| 76 | Eng. | 36 | | M. | 6 | 2 | 1 y | | " " | Chr. metritis. |
| 77 | Irel'd | 21 | | | | | | | " " | Debility. |
| 78 | " | 26 | | M. | 1 | | 10 y | 2 y | " " | Metritis. |
| 79 | Ill. | 8 | School. | | | | | | " 28 | Pleuritis. |
| 80 | Irel'd | 11 | School. | | | | | | " " | Quinsy. |
| [illegible] | City. | 6 m | Babe. | | | | | | " " | Herpes solaris. |

The substance of this report is taken from Prof. Miller's remarks at the clinic. The arrangement is intended to suggest the nature of the cases, the manner of treatment, and the quality of instruction to be had by attendance upon this Department of the Dispensary.

Oct. 20, 1870. *Endometritis and retroversion.* Mrs. C., aged 30, ruddy and corpulent, rendering the introduction of the speculum difficult. She has been wearing a Hodges pessary a week. It will be allowed to remain. Application within the cervix uteri, by means of a probang, armed with a bit of cotton batting, of the following mixture: Solution of the persulphate of tron; compound solution of iodine and glycerine, of each equal parts; carbolic acid added, five grains to the fluid ounce. Two or three probangs prepared, in order to bring the mixture surely in contact with the living tissue.

Oct. 27. The pessary which she has been wearing a fortnight will be removed and left out a week. The indication is to cure the inflammation. Mechanical supports are never to be kept in without careful observation. They should be removed occasionally and medicine reapplied, for it might be that some spot had become irritable. The amount of inflammation has been lessened by the mixture described. Surgeons might use it with good results, getting the ultimate effect of iodine, the astringent effect of iron, and the peculiar effect of glycerine, which is to diminish engorgement. This patient had been on treatment for a long time before coming here, having run the gauntlet of inflammation, retroversion, and other grave conditions. She despaired of ever being any better; but, since her appearance at the Dispensary, she has greatly improved.

Nov. 3. The womb is found to be patulous, and yet in place. There being no displacement appreciable now, the pessary will be left out.

Nov. 10. She will need treatment for some time to come, but we see improvement from week to week. Ordinarily a case like this will give a physician a patient for two years. There is some inflammation yet. She says she has just had her courses and did not see a drop. All the blood in her body rushed up to her head, as she thinks. We will see her a day or two before she is to be unwell again, and will then apply a glycerine pessary.

Oct 20, 1870. *Inflammation of the vulva and cervix uteri.* Extremely irritable. Miss D. The secretion removed not healthy; the cervix excoriated. Nitrate of silver in solid stick, carried directly to the parts affected. This will arrest superficial inflammation as quickly as anything.

Oct. 27. It requires the greatest care and delicacy to introduce the speculum without pain; the very best that can be done causes a little suffering. Cervix exposed. It requires considerable force to remove the mucus. It is of the character of pus. Signs of inflammation are still remaining on the outside of the neck. Nitrate of silver applied. Many practitioners do not succeed because they disregard the fact that the effect of the first application is merely to coagulate the mucus. As the speculum is withdrawn, coming to the vulva we see considerable inflammation about the orifice of the urethra. Parts of the vulva are swollen. Nitrate of silver applied here.

Nov. 3. It has been discovered that there is displacement of the uterus, as well as inflammation of the vagina and vulva. We find heat and swelling. The canal is contracted, therefore, and requires more care than usual in inserting the speculum. Probangs soaked in a solution of nitrate of silver brought in contact with the entire surface. An application within the vulva or near it, is attended with more pain.

Nov. 10. A solution of nitrate of silver, forty grains to the ounce, applied to the vulva and to the vagina.

Oct. 20, 1870. *Cervicitis.* Mrs. G. The os uteri is low, quite within the vulva. The neck is hard and elongated. Whenever there is a tendency to bleed, the mixture of iodine, iron and glycerine is best; it causes no pain, and is very efficient in overcoming inflammation. She has had three children and one miscarriage, but no children for the last eight years.

Oct. 20, 1870. *Ulceration.* Mrs. P. It is necessary to our own comfort to make a digital examination to know the direction and the position of the os uteri. If we should find difficulty with the speculum, it would be best to reintroduce it. It might be necessary to use the sound in order to bring the os uteri perfectly and squarely within the speculum. Cotton used to engage the mucus, which was tenacious and yellowish. Solution of nitrate of silver, five scruples to the ounce of water, applied twice; it is better than the solid stick. The second application acts on the living tissues; compared to the treatment of granular ophthalmia.

Dec. 1. Prof. Miller intentionally put the speculum to the surface of the vagina, and called attention to the fact. He next elevated the os uteri by drawing it up with a sound, while the speculum was withdrawn a little, then put forward, to engage the cervix. This is a typical case of ulceration of the os uteri. The inflammation is limited to the neck. It is cervical metritis, involving both the mucous membrane and the other tissues. It is not sufficient to apply the medicine on the outside of the cervix merely; it should be passed within.

Oct. 20, 1870. *Chronic engorgement, inflammation, and the result of inflammation.* Mrs. McM., aged 35. The os uteri is enlarged. There is a sanious discharge, obscuring the os until it is removed. A slight touch will produce blood Here the ulceration is between the lips. These lips are larger than in health, thickened by inflammation. The mixture of iron, iodine and glycerine applied so as to come in contact with the living tissue. Engorgement simply does not give a correct idea of this case; there is inflammation. It requires a little aid from the sound to bring the anterior lip into view; with a speculum the size of that we are using, it could not all be engaged. There is an increase of tissue, not simply an increase of fluid. Ordinarily nitrate of silver would have been used, but one week of this mixture in such cases is better than four weeks of nitrate of silver. It may be applied every five or six days.

Oct. 27. Before introducing a speculum to bring the os uteri into view, we make a digital examination to find that there is a remarkable roughness and hardness of the os uteri, not scirrhous, but the hardness is due to inflammation, and the roughness to loss of tissue. The speculum passed into the vagina, no attempt to engage more than the anterior lip of the os uteri. The condition here is not so much one of ulceration, to the eye, as it was a week ago. We must remove the hardness. The probang, loaded with cotton saturated with the mixture of iodine, iron and glycerine, passed an inch within the cervical canal. The peculiar effect of the mixture is aided by the carbolic acid, which is one of the most efficient local stimulants and anæsthetics; without it there would be smarting and pain. It always happens, in the use of this mixture, that unless it be made to pass into the cervix instantly, it will not go at all.

Oct. 20, 1870. *Amenorrhœa.* Miss R., aged 23. Her periods were interrupted on her voyage from Ireland to America, three months ago. There is no pain; she complains of a sense of fatigue. There is glossitis. Her bowels are tympanitic. Her feet are swollen. Treatment: a mixture of the saturated solution of chlorate of potash, and the tincture of chloride of iron, to be taken three times daily after food.

Oct. 20, 1870. *Infantile atrophic paralysis.* Willy G., aged eighteen months. He has been subject to chronic diarrhœa, an obvious cause of paralysis. He lost the use of one of his arms four weeks ago. It was sudden—in the morning he could creep, but in the evening he was without the power of moving his arm or his hand in the slightest degree. He can move them freely now, but is still unable to creep. The treatment was a simple diarrhœa mixture and cod liver oil. Chlorate of potash and tincture of chloride of iron have been given three times daily as a tonic.

Oct. 20, 1870. *Prolapsus uteri.* Mrs. D. She has been wearing an instrument which is not quite in place to-day. It will be taken out to reinsert. A longer instrument substituted, in order to hold the womb higher. It is an extreme case.

Oct. 20, 1870. *Unilocular ovarian cyst.* Mrs. A., colored. This patient came for diagnosis. There is no need of exposure. The enlargement began over the right ovary, six or eight months ago. There is no irregularity, no hardness. The enlargement is uniform. We do not suspect pregnancy. The speculum reveals a little appearance of uterine difficulty that ought to be treated. The mouth of the womb is quite irregular, one side of the cervix enlarged, the secretion purulent. The sound proves that the abdominal enlargement is not of the womb. A whalebone probang and clean cotton used to apply the mixture of iron, iodine and glycerine to the cervix uteri. There is by this means no danger from the use of pencils that have been employed in the treatment of other diseased patients. By succussion an impulse on one side is communicated to the opposite side. It is fluid—it is not gas. Is it of peritoneal origin? There is no dullness in the iliac region, not so great at least. Dullness is greatest on the top. There is no Bright's disease; no swelled inferior extremities, to indicate obstructed return of the blood. It is a unilocular or ovarian cyst; a Graaphian follicle has become enlarged. Treatment: for the present, diuretics.

Oct. 20, 1870. *Endocervicitis.* Mrs. C. The disease is not likely to cure itself. There is no doubt of the presence of excoriation, for, on removing the mucus, blood starts. The neck of the womb is also enlarged. In this case our favorite specific application introduced into the canal, has the best effect. The same has been applied to the outside of the cervix also.

Oct. 20, 1870. *Prolapsus uteri and ulceration.* Mrs. D. These very often go together. Here the os is just within the vulva, and it is rough. There is swelling at the orifice of the urethra. The whole vagina is involved in the inflammation. The ulceration has been overcome, but granulation exists. Pass a weak solution of nitrate of silver, to strengthen the parts and to prevent a retrograde. Pass it on to the outside also, for the purpose of producing the peculiar effect of the nitrate externally.

Oct. 27. The cylindrical speculum is preferred on account of haste and the little pain caused by it. The appearance of the cervix before the removal of mucus again exhibited. Cotton by forceps, then by torsion, to entangle the tenacious mucus. Observe the secretion. Inflammation here exists along the canal. The nitrate of silver applied last week caused some hemorrhage. We will apply the mixture of iron, iodine and glycerine to-day. The probang will pass into the cervix nearly an inch, in some cases an inch and a fourth, till it comes to the os internum. Two probangs are required to produce effect. Apply still another outside. A porous plaster ordered to her side, to relieve pain.

Nov. 3. Improving. The roughened cervix looks as though tissue had been lost by inflammation, it is so irregular.

Dec. 1. The cervical neck has a band drawing it to the left side, and front of the pelvis. In removing mucus or blood or whatsoever else it may be, apply force enough to detach it.

Oct. 27, 1870. *Debility.* Mrs. D., aged 38. Complains of cough, and soreness all over, worst especially across the loins. She had not seen her courses for three months, but week before last they returned. She has had seven children; the last, seven years ago. She has been complaining over a year. An ulcer of the cheek communicates with necrosis of bone. She avers that her jaw was broken by a dentist. Treatment: the solution of chlorate of potash and tincture of chloride of iron. Referred to the Surgical Department.

